# Toxicological Impact of Rare Earth Elements (REEs) on the Reproduction and Development of Aquatic Organisms Using Sea Urchins as Biological Models

**DOI:** 10.3390/ijms23052876

**Published:** 2022-03-06

**Authors:** Chiara Martino, Teresa Chianese, Roberto Chiarelli, Maria Carmela Roccheri, Rosaria Scudiero

**Affiliations:** 1 Department of Biological, Chemical and Pharmaceutical Sciences and Technologies (STEBICEF), University of Palermo, Viale delle Scienze, Building 16, 90128 Palermo, Italy; chiaracomlib@yahoo.it (C.M.); roberto.chiarelli@unipa.it (R.C.); maria.roccheri@unipa.it (M.C.R.); 2 Department of Biology, University Federico II, Via Cintia 21, 80126 Napoli, Italy; teresianachianese@libero.it

**Keywords:** calcium uptake, sea urchin embryonic development, gene expression, gadolinium, reproduction

## Abstract

The growing presence of lanthanides in the environment has drawn the attention of the scientific community on their safety and toxicity. The sources of lanthanides in the environment include diagnostic medicine, electronic devices, permanent magnets, etc. Their exponential use and the poor management of waste disposal raise serious concerns about the quality and safety of the ecosystems at a global level. This review focused on the impact of lanthanides in marine organisms on reproductive fitness, fertilization and embryonic development, using the sea urchin as a biological model system. Scientific evidence shows that exposure to lanthanides triggers a wide variety of toxic insults, including reproductive performance, fertilization, redox metabolism, embryogenesis, and regulation of embryonic gene expression. This was thoroughly demonstrated for gadolinium, the most widely used lanthanide in diagnostic medicine, whose uptake in sea urchin embryos occurs in a time- and concentration-dependent manner, correlates with decreased calcium absorption and primarily affects skeletal growth, with incorrect regulation of the skeletal gene regulatory network. The results collected on sea urchin embryos demonstrate a variable sensitivity of the early life stages of different species, highlighting the importance of testing the effects of pollution in different species. The accumulation of lanthanides and their emerging negative effects make risk assessment and consequent legislative intervention on their disposal mandatory.

## 1. Introduction

The aquatic environment is a sink for many anthropogenic contaminants, the diversity of which and their concentrations are rapidly increasing. Therefore, understanding the impact of the human-derived waste on aquatic animal species is a major issue for the scientific community, with a major aim to translate this understanding to global awareness. Despite the relevance of this emergency, few studies have been carried out so far to understand the complex interactions between environmental stressors and their biological effects on the whole ecosystem. A major challenge in impact and risk assessment of environment is to link the harmful effects of pollutants in individual sentinel animals and their ecological consequences.

## 2. Rare Earth Elements

**Figure 1:**
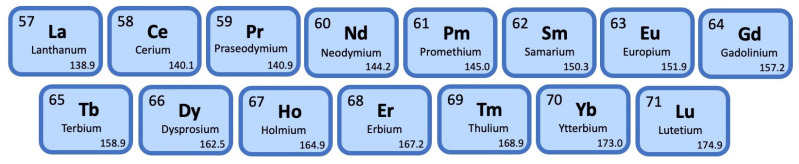
List of the 15 lanthanoid elements with their atomic numbers, symbols and molecular masses.

Among the contaminants of emerging interest, rare earth elements (REEs) play a fundamental role; they consist of a group of 15 trivalent elements of the lanthanide family with atomic numbers of 57 to 71 (IUPAC, 2005) (Figure 1) and are used in various fields of technology such as medicine (diagnostics and drugs), electronic devices (cell phones and plasma screens), and permanent magnets [1].

REE extraction and processing into commercial products have grown exponentially in recent years and have raised environmental concerns about their release into aquatic and terrestrial ecosystems [2,3,4,5,6]. Increased concentrations of REEs have been reported in lakes, rivers, and marine environments, making the removal of REEs from wastewater almost impossible [2,5,7]. The ratio of the concentration of a chemical in biota to that in ambient water is called the bioconcentration factor (BCF) and depends on many parameters; in the case of lanthanides, it depends on the type, the exposure concentration, and the microorganism considered [8]. BCF is a very common parameter to describe the accumulation of pollutants in biota relative to water [9]. It can be expressed as the ratio of the concentration of a chemical in an organism to the concentration of the chemical in the surrounding environment, so BCF is expressed in units of liter per kilogram. The BCF of lanthanides in algae reaches values up to 3,000,000 L/kg [10]. The importance of the chemical mobility of a REE is its link to its bioavailability. In physiological studies, lanthanides are often used to inhibit the uptake of divalent calcium ions (Ca2+, which have a similar ionic radius but a lower charge density than the trivalent lanthanide ion [11,12]. However, in addition to the health risks associated with their applications, ecotoxicological and epidemiological evidence are directly linked to environmental exposure to REEs and adverse health conditions remain weak [2].

## 3. Gadolinium

Among the lanthanides, gadolinium is widely used as a contrasting agent in the diagnostic procedures of magnetic resonance imaging (MRI) due to its paramagnetic properties [13]. For this reason, Gd is becoming a new powerful contaminant of aquatic environments, and the most common pollutant of the lanthanide family [3,14]. Gadolinium, like other REEs, is naturally occurring in the environment as a result of the dissolution of minerals. The background Gd level only minimally affects the total Gd measured in fresh and marine water, since most of the Gd^+3^ ions determined in aquatic environments derive from gadolinium-based contrast agents (GBCAs) excreted via the kidney by MRI patients [15]. GBCAs are eliminated unmetabolized. Being at first generally considered safe, the use of Gd in medicine has grown exponentially in the last decades, leading to a consequent increase in its emission into the environment, although its use has later been linked to nephrogenic systemic fibrosis (NSF) disease [16,17]. Indeed, in 2016, an annual emission of 4 tons of Gd has been estimated in Germany and to 19 tons in Europe [5,7,18]. Increasing concentrations of Gd have been found in rivers, where it passed from the geogenic background of a few ng/L to about 100 µg, with peaks of 1 mg, per liter [5,7,18,19]. High Gd concentrations have also been found in drinking water [3]. An increase in Gd concentrations has also been demonstrated in coastal seawaters, as occurring in Nagoya City near sewage treatment plants [20]; in areas of San Francisco Bay surrounded by hospitals and research centers using GBCA where the Gd concentration passed from 8.27 to 112 pmol kg^−1^ in the last 20 years [21]; and on the of northeastern coast of Brazil, near the city of Salvador, where it is estimated that between 698 and 2021 g Gd per day are discharged into the oceans due to a submarine outfall sewage along the Brazilian coasts [22]. In the future, GBCA concentrations in the aquatic environment are likely to increase due to the constant use of MRI [23].

The presence of Gd has been confirmed in aquatic organisms, such as lichens, algae, plants, invertebrates and vertebrates [3,14,24,25]. However, like other REEs, detailed studies on metabolization, bioaccumulation, and the environmental fate of Gd complexes and their toxicological effects on living organisms are lacking.

In this framework, it is important to develop appropriate methodologies to gain a deeper understanding of the impact of REEs on several important processes, including fertilization and embryonic development. In particular, the use of biological model systems should provide valuable information on this fundamental issue.

## 4. The Sea Urchin Embryo 

The sea urchin (Echinodermata: Echinoidea) embryo has long been used as a model organism for biological developmental studies [26,27,28,29]. Several factors make this system suitable for conducting a wide range of biological tests. These include low maintenance costs; a small size; high fecundity; simple artificial spawning; fertilization and rearing; rapid synchronous development; optical transparency of the embryo, allowing for direct observation of cell division and movement within the embryos and larvae; and well understood embryogenesis. Furthermore, as invertebrate species, sea urchins are not subject to animal welfare concerns. This trait satisfies the strategy for developing alternative approaches to the use of vertebrates in biological testing [27].

Sea urchin embryos have been used successfully to study many cellular processes, such as adhesion [30], differentiation, survival, and death [29,30,31,32,33]. They are also recognized as an excellent model system for eco- and geno-toxicological studies [34,35,36].

Sea urchin embryos are being used as a model to elucidate the role of cellular and molecular mechanisms involved in human health and disease. This is because general cellular properties are common to many organisms [37,38]. Complete sequencing of the sea urchin genome also revealed that sea urchins are more closely related to vertebrates, with which they share the superphylum group of deuterostomes, than other invertebrates that are commonly used as models of human disease, e.g., *Drosophila melanogaster* and *Caenorhabditis elegans* [27].

## 5. Rare Earth Elements and the Sea Urchin Embryo 

Regarding toxicological studies, it is well documented that echinoderm early life stages exhibit a high sensitivity to several toxicants, including heavy metals, persistent organic pollutants and microplastics [39,40,41,42,43,44,45]. The effects of lanthanides on sea urchins have also been evaluated; in particular, the potential damage of seawater contamination on gamete viability, fertilization, and larval development [46,47,48,49,50,51,52,53,54,55]. In these studies, many sea urchin species are utilized, in consideration of their availability in the different areas interested in ecotoxicological studies.

In the *Sphaerechinus granularis* (family Toxopneustidae), a sea urchin family inhabiting the Mediterranean Sea and eastern Atlantic Ocean, sperm exposure to REEs induces an overall inhibition of fertilization success and developmental defects. Of REEs, 10^−5^ M Gd^3+^ ions show the greatest sperm toxicity [55]. Cytogenetic analysis of REE-exposed *S. granularis* embryos reveals the induction of mitotic aberrations to different extents by all tested REEs, with the highest aberration frequencies being induced by 10^−6^ M Gd^3+^. The inhibition of mitotic activity and the frequency of mitotic aberrations were observed in embryos exposed to REEs at concentrations ranging from 10^−6^ to 10^−4^ [54,55].

Interestingly, experiments performed on different sea urchin species demonstrated different REE sensitivities. *S. granularis* displayed significantly greater sensitivity than the other two species with which it shares a habitat, *Arbacia lixula* (family Arbaciidae) and *Paracentrotus lividus* (family Parechinidae), both following embryo and sperm exposure.

In *P. lividus*, sperm exposure to REEs (10^−5^ to 10^−4^ M) resulted in a concentration-related decrease in fertilization success along with increase in offspring damage. Following sperm exposure to 10^−4^ M trivalent REE salts, a significant loss of fertilizing capacity was exhibited by all tested REEs (La, Ce, Nd, Sm, Eu, Dy, Gd, Yb). Eu ions displayed the most severe spermiotoxicity. The induction of transmissible damage presented as developmental defects in the offspring was significantly increased following sperm exposure to REEs, with Yb and La cause the most severe developmental defects. REE-exposed sperm caused offspring showing cytogenetic anomalies and aberrations, along with significant inhibition of mitotic activity. Exposure of *P. lividus* embryos and/or larvae to REE salts in the micromolar range from 10^−6^ to 10^−4^ M determined developmental defects, lipid peroxidation and redox anomalies. The most severe effects were recorded with Gd, followed by Yb, La, Nd, Eu, Ce, and then Sm. Gd and La were also effective in exerting cytogenetic defects [47,49].

Exposures of *A. lixula* spermatozoa to REEs did not affect the fertilization success. On the other hand, the offspring of *A. lixula* REEs-treated spermatozoa suffered significant developmental defects, as recorded in *P. lividus* offspring, particularly after exposure of the spermatozoa to Dy, Er and Yb. These three elements were found to be highly toxic to embryos and larvae. Minor defects were recorded in embryos/larvae exposed to Ce and Lu [47,54].

## 6. Gadolinium and the Sea Urchin Embryos

Considering the massive use of Gd in diagnostics, the toxicity of this REE on embryonic development in sea urchins was particularly studied. The responses to different Gd concentrations during sea urchin development in *A. lixula* and *P. lividus*, two species belonging to the same habitat (north western coast of Sicily, Mediterranean Sea), have been compared with those recorded in two phylogenetically and geographically distant species, *Heliocidaris tuberculata* (family Echinometridae) and *Centrostephanus rodgersii* (family Diadematidae), both from the Pacific Ocean (eastern Australia), that share similar larval phenotypes to theMediterranean species [50]. Indeed, 48 h post fertilization (hpf) these four species go through a feeding echinopluteus larva that show a tripartite gut and a complex three-dimensional skeleton.

These studies retrieved, for the two Mediterranean species, similar values of EC50 (1.18 mM for *P. lividus* and 2.1 mM for *A. lixula*) and an identical NOEC value of 250 nM, whereas the two oceanic species showed great differences in sensitivity. There was a difference of several orders of magnitude in the EC50 and NOEC value between *H. tuberculata* (56 nM and 1 nM, respectively) and *C. rodgersii* (132 mM and 1 mM, respectively) [50]. Based on this, the authors suggested that the different Gd sensitivity shown by the two Australian species may be due to the different phylogenetic history of these sea urchins [50]. Indeed, phylogenetic analysis indicated *C. rodgersii* as the oldest sea urchin lineage among the four examined, and *H. tuberculata*, the most Gd sensitive species, as the more recent and more closely related to the Mediterranean species [56]. Fascinating is the hypothesis that *C. rodgersii* is the most resistant to Gd insult because it is from an older lineage. This feature might have contributed to the adaptive capacity and resilience of *C. rodgersii* in a changing world [57]. However, exposure to Gd led to greater alterations or inhibitions of skeleton growth for the species examined at the final endpoint (48 hpf); the phenotypic response to Gd of the altered skeleton formation was similar in the four species, indicating a similar response mechanism, although with different levels of sensitivity with respect to the concentrations used. Indeed, in all the four species examined, exposure to Gd had no major effect on early development up to gastrulation (24 hpf); the ingression and migration of the PMCs and the invagination of the vegetal plate occurred with the right timing; and at 48 hpf, the differentiation of the ectodermal territories in the columnar epithelium at the animal pole and the in the squamous epithelium at the vegetal pole also appeared normal, as well as the development of the tripartite intestine. On the contrary, at 48 hpf, impaired skeleton development with several skeletal anomalies was evident [50,51].

Similar evidence for a conserved mechanism of Gd toxicity for sea urchin embryos was found in three Japanese species, *Hemicentrotus pulcherrimus*, *Heliocidaris crassispina* and *Pseudocentrotus depressus* [48]. Even in these species the ingression of PMCs into the blastocoel in the late blastula stage, their migration along the inner surface of the blastocoel, and the formation of both ventrolateral clusters at the gastrula stage were not inhibited by Gd-containing seawater; however, the progress of archenteron invagination was accompanied by the formation of abnormal and asymmetrical spicules. The degree of sensitivity to Gd ions also varied in these species. A concentration of 1 µmol/L Gd exerted greater inhibitory effects on spicule formation in *A. crassispina* and *P. depressus* than in *H. pulcherrimus*. Since this Gd level is considered insufficient to block divalent calcium ion (Ca^2+^) channels [58,59], the authors excluded that the inhibition of spicule formation may be caused by the inhibition of Ca^2+^ channels and suggest that trivalent Gd ions exerts their inhibitory effects on spicule formation specifically during the late blastula stage through different molecular pathways, rather than during the gastrula stage when spicule formation effectively occurs.

**Figure 2:**
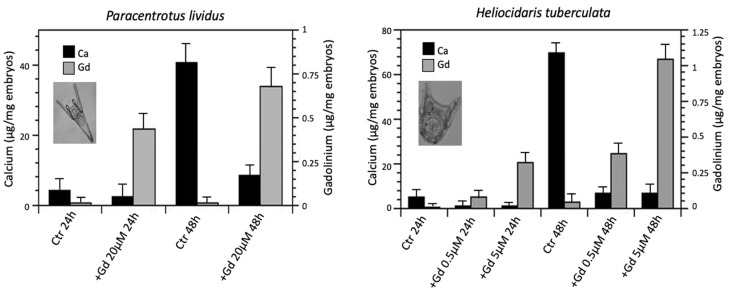
Calcium (Ca) and gadolinium (Gd) content determined by flame atomic absorption spectrometry in *P. lividus* and *H. tuberculata* embryos at 24 and 48 h post fertilization. The bars represent the metal content (mean ± SD) (*n* = 3) determined in embryo pools from three separate fertilizations. Determinations were performed in triplicate. Data were analysed by a one-way analysis of variance (ANOVA).

However, measurements of Ca and Gd content in sea urchin embryos using flame atomic absorption spectrometry (FAAS) clearly demonstrated a correlation between Ca and Gd uptake, with a reduction in the amount of Ca in parallel with an increase in the Gd content [51]. In control embryos, Ca uptake increases significantly during development, the total amount of Ca being 10-fold and 13-fold greater in 48 hpf larvae than in 24 hpf embryos in *P. lividus* and *H. tuberculata*, respectively (Figure 2). It should be considered that the production of spicules in 48 hpf larvae is at its highest, resulting in the formation of a more complex three-dimensional skeleton in *H. tuberculata*. In *P. lividus* embryos developed in Gd-contaminated seawater, there is an approximately 45% and a 78% reduction in the amount of Ca after 24 and 48 hpf, respectively. Gd uptake reaches about 0.70 µg/mg in embryos (Figure 2). 

In *H. tuberculata* embryos exposed to Gd, the reduction in the amount of Ca is about 80% at 24 hpf and 90% at 48 hpf, regardless of the concentration of Gd in water, thus demonstrating that even a low concentration of Gd (0.5 µM) is sufficient to block calcium absorption. Conversely, concentration-dependent uptake of Gd was observed, with a greater Gd amount at the highest concentration (5 µM Gd) at both 24 and 48 hpf (approximately 0.4 and 1 µg/mg embryos, respectively) (Figure 2).

The results showed that Gd interfered with Ca uptake, probably because Gd competes with Ca to bind to the same ion channels. As a trivalent ion, Gd bound with higher affinity than the divalent Ca ions [60]. As expected, the FAAS results also showed that Gd accumulated in sea urchin embryos in a time- and dose-dependent manner. 

The different sensitivity of embryos belonging to different species of sea urchins has also been demonstrated for other contaminants, such as nanoparticles [61], antibiotics, and disinfectants [62]. The different sensitivity to REEs depended on the species considered, but also on the type of lanthanide. Indeed, Gd^3+^ ions induced high severe effects in *P. lividus* embryos than in *S. granularis* and *A. lixula* embryos. This result was in contrast with the findings of the other REEs, where *S. granularis* and *A. lixula* appeared more sensitive than *P. lividus*. Table 1 summarizes the effects of REEs on sperm and embryonic development evaluated on various sea urchin species. This paradoxical finding deserves further investigation. Moreover, it suggests that in studies of biomonitoring, toxicity, and conservation of biodiversity, organisms deriving from different species within the same phyletic group should be used, even when performing a widely consolidated experimental model such as that of the sea urchin embryo.

## 7. Synergistic Effects of Gadolinium Exposure and Increased Temperature on Sea Urchin Embryos

Climate change has severe effects on coastal areas, as global warming has been raising the sea surface temperature since the late 19th century [63]. Understanding the combined effects of water pollution and global warming has therefore become an urgent issue, as this can generate additive, antagonistic, or synergistic effects. The impact of ocean warming and polluting nanoparticles, such as nano zinc oxide (nZnO), was studied on the larvae of the tropical sea urchin, *Tripneustes gratilla*. Both stressors had strong interactive effects on fertilization and development of these larvae. In particular, larval growth was strongly influenced by the combined stress of high temperature and nZnO [64]. More recently, microplastics have been shown to aggravate the effect of an increase in water temperature by generating additional stress on *P. lividus* larvae, manifested in a lower growth rate and impaired development [65]. Regarding a possible combined effect of temperature and lanthanides on sea urchin embryos, to date only one published article focuses on this topic. In this study, the interactive effects of increased temperature and Gd pollution during *P. lividus* development were investigated [53]. Using combined treatments of three temperatures (control: 18 °C; near-future projections: 21 °C; today’s marine heatwave conditions: 24 °C), the authors demonstrated that a lower thermal increase (21 °C) reduced the negative effects of Gd exposure (20 μM) on development, with embryos and larvae having a lower percentage of abnormalities and better skeletal growth, while the higher thermal increase (24 °C) had a negative synergistic effect when combined with Gd exposure, causing a lower percentage of embryos reaching advanced larval stages. At the molecular level, the combined treatments induced the expression of the Hsp60 protein at 24 h, and elicited autophagic and apoptotic processes at 48 h. These apoptotic events were found to be selective, probably to eliminate the most damaged cells. Overall, the authors concluded that a moderate increase in seawater temperature (+3 °C, i.e., 21 °C) will mitigate the negative effects of Gd pollution on embryonic development, while a major increase (+6 °C, i.e., 24 °C) would have negative synergistic effects [53]. 

## 8. Gadolinium and Embryonic Gene Expression in Sea Urchins

The mechanisms underlying the induced toxicity in sea urchin embryos have been investigated by gene expression analyses. In particular, the possible changes in the expression of genes that are involved in the early stages of development, in spiculogenesis, and in cell signaling were determined in embryos of *P. lividus* and *H. tuberculata* exposed to Gd [52].

The choice fell on these two species because, as mentioned before, they exhibit a similar morphological response to Gd, although *H. tuberculata* shows greater sensitivity. In both species, the PMC ingression and archenteron invagination occurred on schedule, but Gd-exposed embryos lacked the two triradiate spicules, thus demonstrating that Gd exposure primarily affects skeleton formation. In both species, some embryos showed supernumerary rods resulting in the inability to achieve the pluteus shape, while others had shorter spicules leading to a reduction in arm length, while still others had an asymmetric skeletal pattern [50]. The developmental success of sea urchin larvae depends on their ability to swim and feed, which is determined by the length of their arms [64]. Therefore, it is likely that Gd exposure can reduces survival in nature. In addition, in both species the uptake of Gd into embryos occurs in a time- and concentration-dependent manner and correlates with the decrease in calcium uptake [52].

Densitometric analysis of the gel bands obtained by the One-Step RT-PCR kit was used by Martino et al. to perform comparative gene expression assays and to detect differences between controls and treated embryos. Starting with an equal amount of total RNA from control and Gd-exposed embryos, they amplified the selected RNA targets reported in Table 2 and the reference genes (ribosomal protein S24 for *P. lividus* and zinc-finger transcription factor Z12 per *H. tuberculata*) used to normalize amplicon band intensities [52]. The authors determined the temporal expression of the genes that are a part of the skeletogenic gene regulatory network, that controls the expression levels of downstream genes involved in mesenchyme differentiation and biomineralization. In particular, they analysed genes expressed earlier during development (e.g., *alx-1*, *nodal*, *lefty*, and *bmp*), and genes involved in molecular signaling and skeletogenesis, such as *univin*, *vegf*, *vegf-r*, *fgf*, *msp130*, *sm30*, *p16*, and *p19*. Table 2 summarizes the expression profiles of these genes in the two species considered, at different concentrations of Gd and hours of development.

*Alx1* is a gene encoding a key transcription factor selectively expressed in the large micromere lineage. It is essential for PMC specification, [66] and the protein regulates the expression levels of many skeletogenic genes, as demonstrated in echinoderm embryos [67,68]. Nodal and bone morphogenetic protein (Bmp) signaling pathways are integral to embryonic axis determination, and their opposite signals regulate left–right asymmetry in the sea urchin larvae [69,70]. *Lefty* (left–right determination factor) expression begins at the 128-cell stage immediately after that of *nodal*, and is rapidly limited to the presumptive oral ectoderm and then shifted to the right side after gastrulation. In the sea urchin embryo, it is essential for the formation of the oral–aboral axis acting as a long-range inhibitor of Nodal signalling [71].

The PCR analysis showed a significant decrease in the mRNA levels of all these genes in Gd-exposed embryos. In particular, *P. lividus* showed the greatest reduction of Alx1 and nodal transcripts which were observed at 48 and 24 hpf, respectively. In *H. tuberculata*, nodal, lefty and bmp transcript levels decreased at 24 hpf, regardless of the Gd dose, whereas at 48 hpf a significant reduction was observed only for the nodal transcripts in embryos exposed to the highest Gd dose.

As for the other genes investigated, it is known that the establishment of a left–right asymmetry in sea urchin embryos involves reciprocal signaling between the ectoderm expressing nodal and a left–right organizer of endodermal origin and requires different signaling pathways including the fgf-erk and univin/vg1 [72], as well as the vegf/vegf-r and fgf pathways, which are essential for providing guidance and differentiation cues to PMCs during sea urchin gastrulation and controlling the directional migration of PMCs and the formation of the embryonic skeleton [73]. The latter is in turn strongly influenced by the expression of the genes encoding the mineralization-related proteins msp130, sm30, p16, and p19 [74].

In *P. lividus* embryos, vegf and vegf-r mRNAs levels were not significantly different to those of controls, either at 24 or 48 hpf, demonstrating that the expression of these signal molecule genes was not affected by Gd exposure. In contrast, in *H. tuberculata*, the vegf expression was significantly up-regulated at 24 hpf, and then reverted to the transcription levels of control embryos at 48 hfp. Significant down-regulation of univin and fgf transcripts was observed in Gd-treated *P. lividus* embryos at 48 hpf. In *H. tuberculate*, the fgf gene expression was down-regulated by Gd treatment, regardless the time and dose of exposure.

Concerning skeletal genes, the analysis performed in *P. lividus* on msp130, p16 and p19 transcripts demonstrated a reduction of the Gd-mediated *msp 130* expression only at 48 hfp, while p16 and p19 Gd-mediated expression increased at 24 hpf and decreased at 48 hpf. In *H. tuberculata* the trend was opposite, as the gene expression of msp 130 responded to Gd only at 24 hpf and returned to control levels at 48 hpf. sm30 gene expression undergoes a Gd-mediated down-regulation during the entire development period.

Although the development of sea urchin larval skeleton requires a conserved and rather complex program of genomic regulation [75], several differences in gene expression have been found in response to Gd treatment in *P. lividus* and *H. tuberculata*. However, it must be considered that these two species are phylogenetically distant and have a structurally different skeleton. *H. tuberculata* produces a robust extended skeleton with fenestrated arm rods, while *P. lividus* produces elongated and thin non-fenestrated rods, typical of Echinidae [76]. This difference could explain the greater amount of calcium in *H. tuberculata* larvae and the different expression of skeletal-specific transcripts between these two species.

Down-regulation of fgf expression in Gd-exposed *P. lividus* embryos at 48 hpf can inhibit spicule elongation. On the other hand, the downregulation of *fgf* observed at 24 hpf and the great up-regulation of *vegf* in Gd-exposed *H. tuberculata* embryos can result in abnormal and excessive branching of the skeleton, as observed in many embryos.

Taken together, the results obtained by studying the harmful effects of Gd exposure on the development of two geographically and phylogenetically distant sea urchin species showed that Gd mainly affected skeletal growth, with an incorrect regulation of the skeletogenic gene regulatory network in both species, albeit with some species-specific differences. Comparative gene expression analysis provided valuable insights to understanding the impact of regulatory changes on the development of differences in specific structures such as the larval skeleton of *P. lividus* and *H. tuberculate*, and the potential mechanisms by which toxic agents such as Gd operate at the genetic and cellular level. The data collected can also provide insight into ecotoxicological issues, to better understand how evolutionary differences affect sensitivity to environmental pollution [52].

## 9. Conclusions

Pollution due to the continuous introduction of human-derived contaminants into the marine environment poses a threat to all marine species. Chemical pollutants represent a constant source of evolutionary challenges for living organisms, strenuously eliciting their adaptive potential [77,78]. A comparative investigation of the toxicity associated with REEs in the early life stages of sea urchins provided evidence for different effects of individual REEs. These effects affect processes such as fertilization, redox metabolism, embryogenesis, skeletogenesis, and the regulation of embryonic gene expression. Early damage in life stages, along with redox, cytogenetic anomalies, and dysregulation of gene expression should be the focus of future REE toxicity studies, as these factors have a major impact on wildlife survival.

It is known that the phenotypic responses to environmental changes can be a key factor in species survival, buffering developmental processes via altered morphology, gene expression, and stress response mechanisms [79,80]. The results demonstrate a variable sensitivity in the early life stages of different sea urchin species, highlighting the importance of testing the effects of pollution for risk assessment in different species, even within the same taxonomic group [48,50,61].

Paradigmatic, as far as sea urchins are concerned, is the case of *S. granularis*, whose greater sensitivity to REEs could indicate a more general sensitivity of this species to environmental pollution, given the notable decrease in *S. granularis* populations observed in the last years in the Mediterranean Sea.

## Figures and Tables

**Table 1:** Toxic effects of REEs on sperm and embryonic development evaluated on various sea urchin species.

Sea Urchin Species	Observed Alteration	REE(s) Concentration	Reference
*S. granularis*	Sperm toxicity Developmental defects	10 µM Gd; 100µM La, Nd, Eu 1–100 µM Yb, La, Ce, Nd, Sm, Eu	[54] [55]
*P. lividus*	Sperm toxicity Impaired larval skeletogenesis Developmental defects	100 µM La, Ce, Nd, Sm, Eu, Dy, Gd, Yb 20 µM Gd 1–100 µM Ho, Gd, Yb, La, Nd, Eu, Ce, Sm	[49] [47] [50,51,52]
*A. lixula*	Sperm toxicity Developmental defects	100 µM La, Sm, Eu 1–100 µM Yb, La, Ce, Nd, Sm, Eu	[47] [54] [50]
*H. pulcherrimus*	Impaired larval skeletogenesis	3 µM Gd	[48]
*A. crassispina*	Impaired larval skeletogenesis	1 µM Gd	[48]
*P. depressus*	Impaired larval skeletogenesis	1 µM Gd	[48]
*H. tuberculata*	Impaired larval skeletogenesis	0.5 µM Gd	[50,52]
*C. rodgersii*	Impaired larval skeletogenesis	150 µM Gd	[50]

**Table 2:** Variations (in percent) of gene expression between control and Gd-treated sea urchin embryos.

Gene Name	Gd-Interference in Expression of Embryonic Skeletogenic Gene Regulatory Network					
	*Paracentrotus lividus*		*Heliocidaris tuberculata*			
	20 µM Gd		0.5 µM Gd		5 µM Gd	
	**24 hpf**	**48 hpf**	**24 hpf**	**48 hpf**	**24 hpf**	**48 hpf**
* alx-1 *	−43 ± 0.07%	−57 ± 0.06%	n.d	n.d.	n.d.	n.d.
* nodal *	−59 ± 0.07%	−20 ± 0.08%	−33 ± 0.01%	=	−33 ± 0.04%	−20 ± 0.01%
* lefty *	n.d.	n.d.	−29 ± 0.04%	=	−43 ± 0.11%	=
* bmp *	n.d.	n.d.	−19 ± 0.05%	=	−29 ± 0.09%	=
* univin *	=	−48 ± 0.05%	n.d.	n.d.	n.d.	n.d.
* vegf *	=	=	+100 ± 8%	=	+179 ± 15%	=
* vegf-r *	=	=	n.d.	n.d.	n.d.	n.d.
* fgf *	=	−58 ± 0.04%	−24 ± 0.03%	−30 ± 0.09%	−19 ± 0.08%	−30 ± 0.1%
* msp130 *	=	−52 ± 0.12%	−34 ± 0.07%	=	−30 ± 0.01%	=
* sm30 *	n.d	n.d	−37 ± 0.06%	=	−35 ± 0.06%	−29 ± 0.03%
* p16 *	−28 ± 0.05%	−49 ± 0.06%	n.d.	n.d.	n.d.	n.d.
* p19 *	+35 ± 0.08%	−23 ± 0.05%	n.d.	n.d.	n.d.	n.d.

Legends: =, unchanged; n.d., not determined. The results are the mean of 9 independent reactions ± SD (from embryos harvested from 9 wells) for the two developmental endpoints. Data were analysed by ANOVA, Levene’s test, and post-hoc Tukey’s HSD test. All the variations were significant (*p* < 0.05).

## Data Availability

Data supporting the findings of this study are available within the article.
